# Yes, it turns: experimental evidence of pearl rotation during its formation

**DOI:** 10.1098/rsos.150144

**Published:** 2015-07-15

**Authors:** Yannick Gueguen, Yann Czorlich, Max Mastail, Bruno Le Tohic, Didier Defay, Pierre Lyonnard, Damien Marigliano, Jean-Pierre Gauthier, Hubert Bari, Cedrik Lo, Sébastien Chabrier, Gilles Le Moullac

**Affiliations:** 1 Ifremer, UMR 241 EIO, UPF-ILM-IRD, Labex Corail, BP 7004, 98719 Taravao, French Polynesia; 2 Ifremer, UMR 5244 IHPE, UPVD, CNRS, Université de Montpellier, CC 80, 34095 Montpellier, France; 3 Ifremer, Centre Atlantique, Rue de l'Ile d'Yeu, BP 21105, 44311 Nantes Cedex 03, France; 4 Véga Industrie, 12 avenue Maurice Thorez, 94200 Ivry sur Seine, France; 5 Laboratoires ICP-TEXINFINE, 60 rue Duguesclin, 69006 Lyon, France; 6 Pearl and Jewellery Museum, Qatar Museums, Doha, Qatar; 7 Direction des Ressources Marines et Minières, BP 20, 98713 Papeete, Tahiti, French Polynesia; 8 Université de Polynésie Française, laboratoire GEPASUD, BP 6570, 98702 Faa'a, Tahiti, French Polynesia

**Keywords:** pearl oyster, pearl rotation, biomineralization

## Abstract

Cultured pearls are human creations formed by inserting a nucleus and a small piece of mantle tissue into a living shelled mollusc, usually a pearl oyster. Although many pearl observations intuitively suggest a possible rotation of the nucleated pearl inside the oyster, no experimental demonstration of such a movement has ever been done. This can be explained by the difficulty of observation of such a phenomenon in the tissues of a living animal. To investigate this question of pearl rotation, a magnetometer system was specifically engineered to register magnetic field variations with magnetic sensors from movements of a magnetic nucleus inserted in the pearl oyster. We demonstrated that a continuous movement of the nucleus inside the oyster starts after a minimum of 40 days post-grafting and continues until the pearl harvest. We measured a mean angular speed of 1.27° min^−1^ calculated for four different oysters. Rotation variability was observed among oysters and may be correlated to pearl shape and defects. Nature's ability to generate so amazingly complex structures like a pearl has delivered one of its secrets.

## 1. Introduction

**Figure 1.**
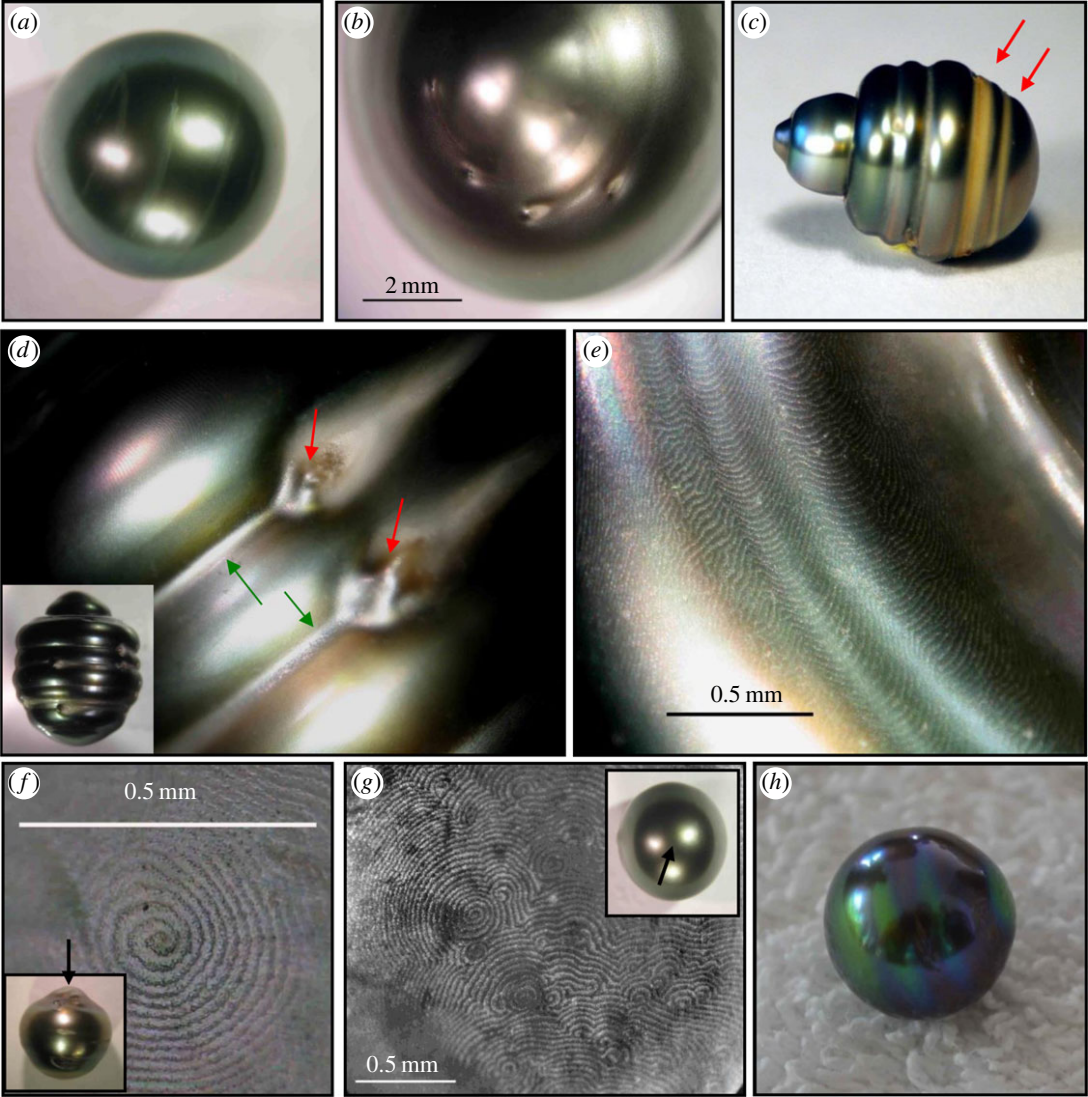
Selection of visual examinations of nucleated cultured pearls suggesting a rotation of the pearl inside the pearl sac of the pearl oyster during its formation. (*a*) Pearl with a smear of dried organic matter deposit extended in coaxial arcs. (*b*) Pearl showing on its surface defects corresponding to holes extended with arcuate and coaxial comets. The arcs of the comets are all directed in the same direction. (*c*) Pearl with multiple and coaxial circles. Red arrows indicate groove filled with ring-shaped organic matter. (*d*) Enlargement of a circled pearl. Observation suggests that defects (red arrows) on the pearl surface have generated, under the effect of rotation, lateral beads separated by a groove (green arrows). The entire pearl is shown in the inset. (*e*) The observation of the same pearl (shown in (*d*)) at high magnification shows the scallop-shaped form of the growing steps of the nacre layer under the effect of the rotation. (*f*) Observation of the spiral-shaped growing steps of the nacre layer at the top of a pearl with axial symmetry. The entire pearl is shown in the inset and the arrow indicates the observation area. (*g*) Observation of a group of spiral-shaped growing steps of the nacre layer at the surface of a hemispherical pearl. The entire pearl is shown in the inset and the arrow indicates the observation area. (*h*) Pearl with coloured rings at its surface suggesting a rotation axis during nacre deposition.

Pearls are the only unique gems produced by a living organism [1]. Their formation process, either by natural means or through human intervention, is a response to an injury to the mantle tissue or a protection against an aggression or an intrusion by a foreign body. The formation of a natural pearl is the result of an accidental transfer of cells of the upper epithelium layer of the mantle into the connective tissue where they multiply to form a closed cyst called a pearl sac [1,2]. Nowadays, pearls are cultured by introducing a nucleus into an animal and biomineral is secreted around it. There are several types of cultured pearls. Non-nucleated cultured pearls are usually mantle-grown in freshwater mussels, whereas nucleated cultured pearls are usually gonad-grown in saltwater oysters [3]. Most nucleated pearl production is from *Pinctada* species and is the product of grafting and then rearing these oysters in their natural environment. The grafting process takes place following a surgical operation during which the graft, a small piece of mantle tissue, is inserted into the gonad of the receiving oyster together with a shell-based nucleus. Once inserted into the receiving oyster, the external epithelial cells of the graft multiply to form a pearl sac, a single epithelium, around the nucleus [3–6]. The pearl sac then starts to deposit calcium carbonate polymorph layers onto the nucleus. This is the starting point of the future pearl (figure 1*a*−*h*). The nacreous layer is composed of aragonite tablets surrounded by an organic framework (chitin and matrix proteins) [2]. Each mineral tablet thickness is of the order of submicrometres. The assembly of mineral and organic components is guided by enzymatic and by self-assembly mechanisms, which are not well understood. A rearing period of more than 1 year is needed to produce finally a pearl with a sufficiently thick layer of nacre before marketing. The minimum nacre thickness allowed for marketing is 0.8 mm.

The last 5 years have seen a rise in studies of nacre formation conducted in the combined disciplines of biology, physics, chemistry and crystallography with the aim to extend our knowledge about pearl [4,7,8] and shell [7,9–13] formation. However, our fundamental understanding of nacre formation remains limited. Few studies are available that explain the pearl mineralization process as well as the formation of defects [14]. A remarkable anomaly on the pearl surface is the presence of circles (figure 1), which intuitively suggests a possible rotation of the nucleated pearl inside the pearl sac of the oyster. Rotation of the nucleated pearl during calcium carbonate polymorph layer deposition around the nucleus is also suggested by various observations. These visual examinations can be done at different levels such as the defects on the surface of the pearl, the steps of growth of nacre layer and more exceptionally with the colour (figure 1). Although these observations strongly suggest nucleated pearl rotation, no characterization of such a movement has ever been described. This can be explained by the difficulty of observation of such a phenomenon in the tissues of a living animal. Evidence of pearl rotation is found in both natural and cultured pearls. It is a universal reality that has never been demonstrated and measured. The phenomenon has been mentioned in a few books or local magazines for pearl farmers, written by the early witnesses of pearl culture in Tahiti [15]. They clearly point to rotation as a necessary explanation for the rings and the pear shapes of Tahitian pearls. More recently, Cartwright *et al.* [14] suggested, based on microscopy observations of pearls from *Pteria sterna* and *Pinctada margaritifera*, a general physical framework for pearl rotation. They argued that pearl rotation is a self-organized phenomenon caused and sustained by physical forces from the growth fronts, and that rotating pearls are an example of a natural ratchet. However, there is no accurate characterization of pearl rotation inside the animal.

## 2. Material and methods

### 2.1 Experimental grafts

We implemented two experimental grafts with a professional grafter. The first was performed on 12 April 2012 with 15 magnetic nuclei of 6.66 mm diameter (electronic supplementary material, figure S1), 15 ‘receiving’ *Pinctada margaritifera* pearl oysters coming from Takarao Island (Tuamotu, French Polynesia) and one ‘donor’ oyster. After grafting, the ‘receiving’ oysters were individually maintained in a pocket of a plastic net, as done by pearl farmers, and hung up on a long line in the experimental oyster growing field of Ifremer in the lagoon of Vairao (Tahiti Island). Nucleus rejection was checked every week post-grafting and only receiving oysters that retained their nucleus were kept. After four weeks, oysters were transferred to the oysters' nursery of Ifremer in raceways with continuous flow of seawater and food (microalgae *Isochrysis galbana*). The first tests on the monitoring system were done 20 days post-grafting. The second graft was performed on 31 May 2012. Thirteen ‘receiving’ oysters from the Ifremer growing field were grafted using four different ‘donor’ oysters and the same nucleus size. After grafting, the ‘receiving’ oysters were individually maintained in the lagoon of Vairao as was done for the first graft. After 12 days, oysters were transferred to the oysters' nursery of Ifremer and the first data acquisitions started at a post-grafting time of day+13.

### 2.2 Data acquisitions experiment

The so-called ‘magnetometer’ system (electronic supplementary material, figures S2 and S3) was specifically engineered for our study to register magnetic field variations with magnetic sensors from movements of a magnetic homemade nucleus (electronic supplementary material, figure S1) inserted in the gonad of a pearl oyster. The magnetometer is made of three connected main parts as described in the electronic supplementary material, figure S2. For the experiments, a rearing tank basin (1×1×0.3 m) has been built with a circular hole of 20 cm in diameter at the centre of the bottom face. The dome comes by below to close the hole (electronic supplementary material, figure S4). During data acquisition, the grafted oyster is placed into the dome in horizontal or vertical position. The tank is filled with an open circuit of seawater. The water level in the basin is about 20 cm and the overflow floods by a spillway. The seawater flow is maintained between 1200 and 1600 ml min^−1^, for a theoretical renewal time of about 3 h 40 min. A tank with the grown microalgae *Isochrysis galbana* (concentration 1500–4000 cells ml^−1^) and seawater is used to feed the oyster at a constant flow of 10 ml min^−1^. Our different times of acquisition varied from 1 to 50 days. The frequency of acquisition is every half-second with a recording periodicity equal to 1 min.

### 2.3 Mathematical treatment of the data

Vectors associated with magnet positions over time are deduced from the magnetic flux density measured at each sensor using (electronic supplementary material, figure S3)

$$\text{Pi}=\overset{25}{\underset{j=1}{\sum }}a_{ij}\times P_{j},$$

where ***Pi*** is the position of the magnet at time *i* in three-dimensional Cartesian coordinates (*x*_*i*_, *y*_*i*_, *z*_*i*_); *j*, sensor number, from 1 to 25; *a*_*ij*_, magnetic flux density (tesla) from the sensor *j* at time *i*; *P*_*j*_, Cartesian coordinates for the sensor *j* on the dome.

The time series obtained were linearly filtered using a centred moving average to remove white noise. Durbin–Watson test was computed to test absence of autocorrelation in residuals (*α*=0.05) and to adapt the size of the window width used while filtering. The radius (*r*) and the coordinates of the centre O (*a*, *b*, *c*) of the best-fit sphere to the set of data points were determined using least-squares estimation from sphere equation:

$$R^{2}={\left(x-a\right)}^{2}+{\left(y-b\right)}^{2}+{\left(z-c\right)}^{2},$$

which correspond to

$$(x^{2}+y^{2}+z^{2})+x.A_{1}+y.A_{2}+Z.A_{3}+A_{0}=0,$$

with *A*_0_=*a*^2^+*b*^2^+*c*^2^−*R*^2^; *A*_1_=−2*a*; *A*_2_=−2*b*; *A*_3_=−2*c*; and $R=\sqrt{a^{2}+b^{2}+c^{2}-A_{0}}$.

Thus, we must solve

$$\left(\begin{array}{c} A_{0}\\ A_{1}\\ A_{2}\\ A_{3} \end{array}\right)={\left(\begin{array}{cccc} n & \sum_{i=1}^{n}x_{i} & \sum_{i=1}^{n}y_{i} & \sum_{i=1}^{n}z_{i}\\ \sum_{i=1}^{n}x_{i} & \sum_{i=1}^{n}x_{i}^{2} & \sum_{i=1}^{n}x_{i}.y_{i} & \sum_{i=1}^{n}x_{i}.z_{i}\\ \sum_{i=1}^{n}y_{i} & \sum_{i=1}^{n}x_{i}.y_{i} & \sum_{i=1}^{n}y_{i}^{2} & \sum_{i=1}^{n}y_{i}.z_{i}\\ \sum_{i=1}^{n}z_{i} & \sum_{i=1}^{n}x_{i}.z_{i} & \sum_{i=1}^{n}y_{i}.z_{i} & \sum_{i=1}^{n}z_{i}^{2} \end{array}\right)}^{-1}\left(\begin{array}{c} \sum_{i=1}^{n}\rho_{i}^{2}\\ \sum_{i=1}^{n}\rho_{i}^{2}.x_{i}\\ \sum_{i=1}^{n}\rho_{i}^{2}.y_{i}\\ \sum_{i=1}^{n}\rho_{i}^{2}.z_{i} \end{array}\right),$$

with *ρ*_*i*_=*x*^2^+*y*^2^+*z*^2^ and *n* corresponding to the number of values *i*.

The sphere centre O was then shifted at the origin. A central projection of vectors onto the sphere surface was applied for correcting remaining error measurements:

$$(x_{i^{\prime }},y,z_{i}^{\prime })=\frac{r}{\left|R_{i}\right|}\times (x_{i},y_{i},z_{i}),$$

with |*R*_*i*_| corresponding to the length of each vector ${\text{OP}}_{i}:|R_{i}|=\sqrt{x^{2}+y^{2}+z^{2}}$.

Eventual movement of the nucleus during the recording period was thus graphically represented by a set of points lying on a unit-sphere surface.

Angles ***Ψ***_*i*_ between successive vectors were then calculated assuming absence of nucleus translation. The axis of rotation was dynamically found every minute using a centred windows size of 130 min, from plane equation using least squares. Thereafter, points were orthogonally projected on the perpendicular plane passing through zero (electronic supplementary material, figure S6). The angle ***Ψ***_1_ between vectors ***OP1*** and ***OP2*** with respective coordinates (*x*_1_, *y*_1_, *z*_1_) and (*x*_2_, *y*_2_, *z*_2_) was thus given by

$$\Psi_{1}=\text{arccos}\;(x_{1}.x_{2}+y_{1}.y_{2}+z_{1}.z_{2}).$$

Angular speed was then deduced by averaging angles ***Ψ*** over time of recording. Angular velocities were estimated only for grafted nuclei rotating around axes changing slightly over time.

### 2.4 Experimental validation of the measurement system

To validate the magnetometer system, we analysed and characterized the artificial movements of the magnet. We used a clock mechanism to get a constant rotation with a known speed. The clock system was put up to the monitoring dome with a stick glued to the minute hand mechanism (electronic supplementary material, figure S7A). The stick was long enough to place a magnet (the same as the ones implanted in the grafted nucleus) in the monitoring area. The rotation axis was the stick axis with a speed of 0.5° min^−1^ set by the hour hand rotation. The first set of experiments was dedicated to data collection and graphical representations of rotations with different rotational axis of the magnet. The stick axis was fixed with an angle varying from 0° to 90° to the horizontal (total recording duration of 8995 min). The magnet was either perpendicular to the stick or once fixed with an angle of 45° (slanted magnet). The magnetic flux of a motionless magnet horizontally positioned was also measured four times to understand better the magnetometer precision in absence of movement (total duration of 5126 min).

The three-dimensional plots of rotating and motionless magnets are presented in the electronic supplementary material, figure S7B–C. Results of angular speed calculation and radius of the detected spheres are presented in the electronic supplementary material, table S1, for the rotating magnets. Shapes of unfiltered points representing the movements of the rotating magnets over time were laid on a sphere surface. After filtering, points were aligned and represented well the rotation on the hour hand (electronic supplementary material, figure S7B). Conversely, unfiltered points describing positions of the motionless magnet over time formed clouds without particular pattern (electronic supplementary material, figure S7C). In this case, the best-fit spheres for the four recordings were always small (mean radius of 0.15±0.13, with a maximum size at 0.36) in comparison with rotating magnets (mean radius of 4.75±2.31, electronic supplementary material, table S1) and pearls (mean radius of 9.26±1.74, with a minimum at 6.31, *N*=15). Points were close together and small position variations were only due to noise. Motionless nucleus could thus be detected using size of the best-fit sphere radius and shape of points. For rotating magnet, angular speed is well approached by the calculations (electronic supplementary material, table S1).

## 3. Results and discussion

**Figure 2.**
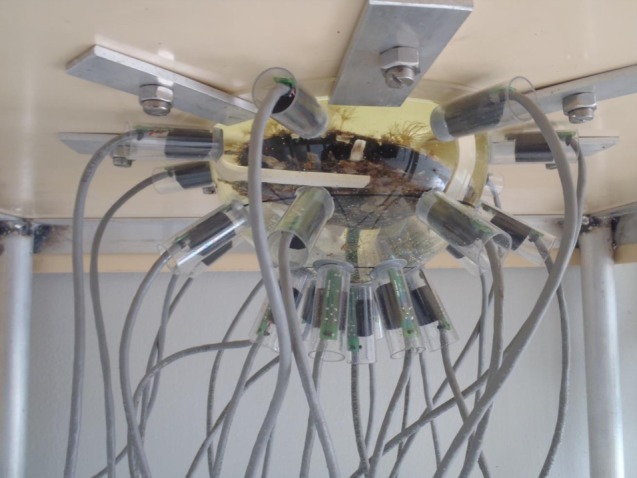
Overview of the magnetometer system. For data acquisition, the grafted pearl oyster *Pinctada margaritifera* is placed into the dome. The movements of a magnetic homemade nucleus (electronic supplementary material, figure S1) inserted in the gonad of a pearl oyster are registered through magnetic field variations measurement with the 25 magnetic sensors located on the convex surface of the dome. The frequency of acquisition is every half-second with a recording periodicity equal to 1 min. Time of acquisition varied from one day to one week. Above the dome, the tank is filled with an open circuit of seawater with the grown microalgae *Isochrysis galbana*(concentration 1500–4000 cells ml^−1^) to feed the oyster at a constant flow of 10 ml min^−1^.

**Figure 3.**
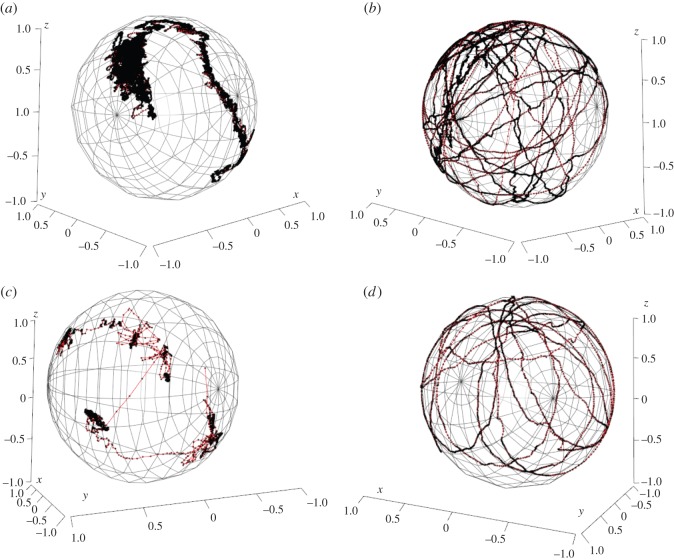
Three-dimensional representation of the nucleus movement in two pearl oysters *Pinctada margaritifera.* Pearl oyster 1: (*a*) from 1 to 40 days after grafting, (*b*) from 40 to 50 days after grafting. Pearl oyster 2: (*c*) from 23 to 24 days after grafting, (*d*) from 30 to 32 days after grafting. Points representing measure of magnet position each minute are linked to red lines to show evolution with time. After a first period of around 30–40 days (*a*,*c*), the nucleus rotation of the two oysters changed and became continuous for the recording periods (*b*,*d*).

To investigate the characterization of pearl rotation, the ‘magnetometer’ system (figure 2 and electronic supplementary material, figures S1, S2, S3 and S4) was specifically engineered to register magnetic field variations with magnetic sensors from movements of a magnetic homemade nucleus inserted in the gonad of a pearl oyster (electronic supplementary material, figure S1). Two oysters were placed under the ‘magnetometer’ system from the first to the 50th day after the grafting. This time window corresponds to the period necessary for the establishment of the pearl sac [14]. For each measurement, the movements of the nucleus were recorded; these becoming more and more stable with increasing time (figure 3). Aside from 180° discontinuous rotation of the nucleus observed for the first 12 days, records showed chaotic movements of the nucleus punctuated with motionless period. After these periods of around 30–40 days, the nucleus rotation of the two oysters changed and became continuous for all the recording period (figure 3*b*−*d*). All measurements performed on nine oysters showed continuous movements of the nucleus after a minimum of 40 days post-grafting. Some rotations could be rather stable (electronic supplementary material, figure S5A), whereas others could show important variations of the rotation axis position over time (electronic supplementary material, figure S5B). Angular velocities are also variable among records. We obtained a mean angular speed of 1.27° min^−1^ calculated for four different oysters, with a maximum mean per recording period of 2.10±0.82° min^−1^ (s.d.) and a minimum of 0.40±0.15° min^−1^ (s.d.). Under assumption of rotations around a fixed axis, these mean velocities correspond to, respectively, one turn in 4 h and 43 min for the mean calculated on four different oysters and 2 h and 51 min and 15 h for extremes.

**Figure 4.**
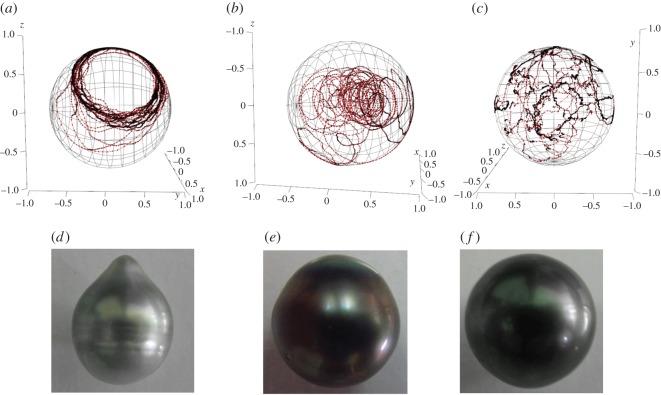
Three-dimensional representation of the nucleus movement for: (*a*) oyster 3 from 881 to 885 days after grafting, (*b*) oyster 5 from 909 to 911 days after grafting and (*c*) oyster 4 from 886 to 890 days after grafting before harvesting their respective pearls (*d*–*f*). Circular motions give an indication of distance between rotation axis and magnet, similar radius indicating conservation of this distance even if circles are localized at different positions on the sphere (i.e. (*c*)).

Rotation variability may be correlated to pearl shape and defects. The last records, on nucleus movement before harvesting, revealed three kinds of pearls for three contrasted rotations (figure 4). Rotations around an axis quite stable in time without additional movements inside the pearl sac were associated with a slightly circled pearl with an axis of rotational symmetry (figure 4*a*−*d*). Rotations in a preferential zone due to slight displacements of the nucleus in the pearl sac between rotations led to a semi-round pearl with one coloured circle on the top (figure 4*b*−*e*). Irregular rotations, with a variable axis position over time and different senses of rotation, gave a non-circled round pearl with consistent colour (figure 4*c*−*f*). According to the hypothesis of Cartwright *et al*. [14] concerning rotation induced by pearl growth, steps of round pearls would not be necessarily randomly oriented across its surface but could globally share a direction varying over time. Rotation in an irregular way could favour homogeneity of deposits on nucleus creating a round pearl. Here, we experimentally showed that the movement of a round pearl is in several successive directions (figure 4*c*−*f*), thereby validating the hypothesis of Cartwright *et al*. [14] which said that for a spherical bead, the movement would be random, while for a pearl with rings (figure 1*c*–*f*), movements would be with a single rotation axis (figure 4*a*−*d*). This is consistent with our observations. Our results are in accordance with the work of Cartwright *et al.* [14] bringing the experimental evidence of the theoretical model.

At the end of 40 days post-grafting, the rotation of the pearl appears to be continuous throughout its complete formation, which lasts 12–18 months. This time window corresponds to the period necessary for the establishment of the pearl sac [14]. The secretion of the pearl sac, which is a single epithelium, is totally dependent on the physiology of the animal. These cells, programmed to secrete the components of the shell, begin to deposit material on the pearl nucleus very soon after the graft. The secretion mechanism, caused and sustained by physical forces from the growth fronts, reproduces the structure of the shell in the pearl and was proposed to be responsible for the pearl rotation [14]. As long as the pearl remains in the animal, the pearl sac continues to secrete nacre-forming compounds; nacre lamellae progressively cover the nucleus, so the pearl grows, turns and the pearl sac expands with it.

## 4. Conclusion

Cultured pearls are human creations formed by inserting a nucleus and a small piece of mantle tissue into a living shelled mollusc, usually a pearl oyster. Then, the biomineral is secreted by the external epithelial cells of the mantle tissue around the nucleus to produce the pearl (figure 1). We have demonstrated here that, during this process, the pearl rotates continuously inside the oyster throughout its formation and we have quantified its movement. We showed that a continuous movement of the nucleus inside the oyster starts after a minimum of 40 days post-grafting and continues until the pearl harvest 12–18 months later. Nature's ability to generate so amazingly complex structures like a pearl is impressive and still partially unknown. One of its secrets has been delivered through this study. Such a challenge will strongly encourage the community to continue to develop approaches and knowledge to decipher the biophysical and molecular details of biomineralization processes.

## Supplementary Material

YG_ROAYLB_version_2403215 ESM__01_06_2015.docx

## Supplementary Material

YG_ROAYLB_version_2403215 ESM__01_06_2015.pdf

## Data accessibility

Raw data are available in the electronic supplementary material.
