# Supplementary material for: Yes, it turns: experimental evidence of pearl rotation during its formation
Source: R Soc Open Sci. 2015 Jul 15;2(7):150144. doi: 10.1098/rsos.150144 (PMC4632584; doi:10.1098/rsos.150144)
Supplement: YG_ROAYLB_version_2403215 ESM__01_06_2015.docx [file rsos150144supp1.docx]

**Supporting information**

**Table S1: Radius of the detected spheres and angular speed of rotating magnets according to angle between rotation axis and horizontal**

|  | 30° axis | 45 ° axis | 60° axis | 90°v* axis | Average± SD |  |
| --- | --- | --- | --- | --- | --- | --- |
| Radius of the detected sphere | 1.69 | 4.53 | 5.64 | 7.15 | 4.75 ± 2.31 | |
| Mean rotation angle before projection (dg/min) | 0.266 | 0.360 | 0.420 | 0.505 | 0.388 ± 0.10 | |
| Mean rotation angle (dg/min) | 0.499 | 0.500 | 0.500 | 0.502 | 0.500 ± 1.26 10^-3^ | |

*v= vertical

**A B**


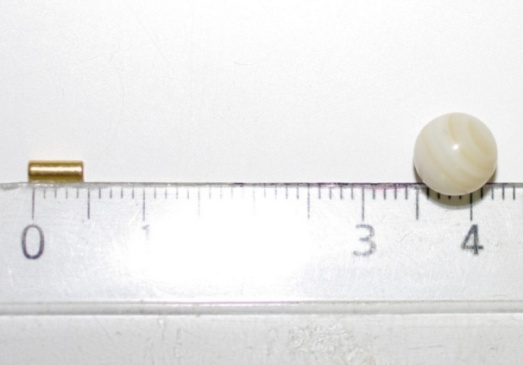

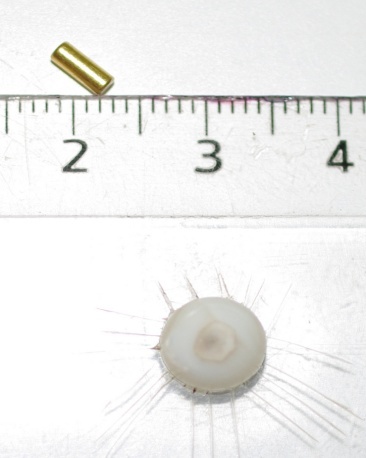


**Figure S1: Magnetic nucleus description.** (A) The nucleus used in our experiment are spherical nacre bead made from the shell of *Amblema sp*. freshwater mussel and were commercially purchased. The choice of the magnet is focused on a neodymium magnet (material: cylindrical NdFeB and length 5 mm). The direction of magnetisation is axial and the magnetising force is N52. These 6.66 mm diameter nucleus were drilled through and a cylindrical magnet of 5mm length was implanted into each of them. The magnet was inserted so that its gravity centre was situated at the centre of the nucleus. (B) After drilling and magnet insertion, dental resin was used to fill the hole. Then, it was polished before grafting to avoid ruggedness.

**
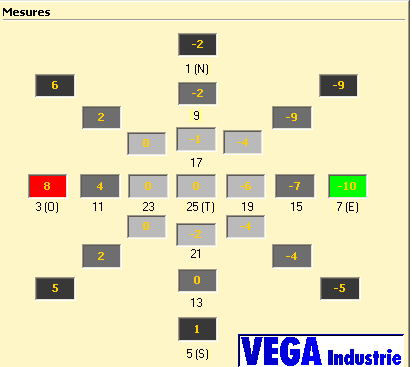

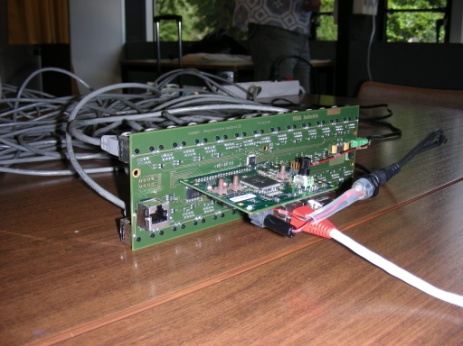

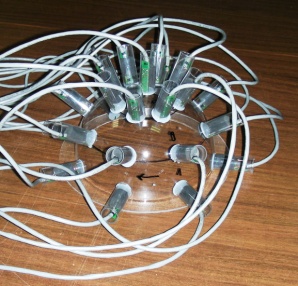
A B C**

**Figure S2: The magnetometer is made of three main parts connected together.** **A.** The measuring part is a dome with sensors. The dome is a half-sphere made of plexiglas (diameter of 20 cm). 25 magnetic sensors with two components, the HMC1021 compass from Honeywell (a one-axis magnetic sensor) and an offset compensation circuit. Twenty-four of the 25 sensors are shared out on the convex surface of the dome as three circles of eight elements at 0°, 30° and 60° of angle with the base. The last one is located on top of the dome at 90°. Each sensor was glued on the dome with a cyanolite paste and is protected from impacts and water by a plexiglas tube. **B.** The electrical part is composed of a data acquisition board with 26 RJ45 female plugs (25 plugs are used to connect the cables from the sensors and the last one carries out the ethernet connection to the computer) and 25 wiring cables, each of them ending with a RJ45 male plug. **C.** The Human Machine Interface (HMI) is a software called “magneto” by *VEGA Industrie*. It is composed of two parts. The first is a microcontroller that uses internal software to collect, process and transfer data to the second part of the HMI. This second part, hosted by the computer, is the computer software (*magneto- magnetometer interface 1.0*). It collects data from the microcontroller, allows real-time sensors and gains data visualisation, tracking the acquisition process. This interface is used to define communication, data acquisition parameters and backup. The following parameters have been defined for our experiments: acquisition frequency: 50 (1/10s); Filtration rate: 5; Recording periodicity: 1 minute. The export data file is a .CSV file, named by the software with references to the date and hour of start: *Magneto_AAAA_MM_JJ_HH_MM.csv.*

**A** **B**


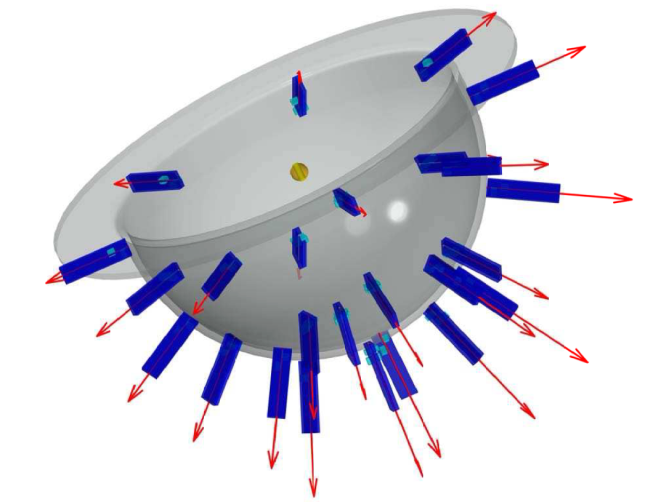

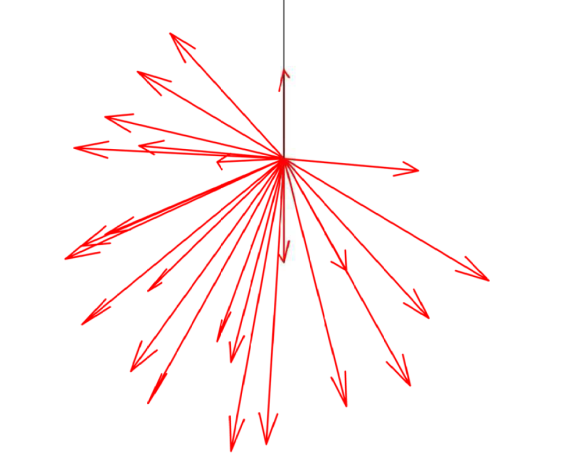


C

| CAPTEUR | 1 | 2 | 3 | 4 | 5 | 6 | 7 | 8 |
| --- | --- | --- | --- | --- | --- | --- | --- | --- |
| X | 1 | +√2/2 | 0 | -√2/2 | -1 | -√2/2 | 0 | +√2/2 |
| Y | 0 | +√2/2 | 1 | +√2/2 | 0 | -√2/2 | -1 | -√2/2 |
| Z | 0 | 0 | 0 | 0 | 0 | 0 | 0 | 0 |
| CAPTEUR | 9 | 10 | 11 | 12 | 13 | 14 | 15 | 16 |
| X | +√3/2 | +√6/4 | 0 | -√6/4 | -√3/2 | -√6/4 | 0 | +√6/4 |
| Y | 0 | +√6/4 | +√3/2 | +√6/4 | 0 | -√6/4 | -√3/2 | -√6/4 |
| Z | 1/2 | 1/2 | 1/2 | 1/2 | 1/2 | 1/2 | 1/2 | 1/2 |
| CAPTEUR | 17 | 18 | 19 | 20 | 21 | 22 | 23 | 24 |
| X | 1/2 | +√2/4 | 0 | -√2/4 | -1/2 | -√2/4 | 0 | +√2/4 |
| Y | 0 | +√2/4 | 1/2 | +√2/4 | 0 | -√2/4 | -1/2 | -√2/4 |
| Z | √3/2 | √3/2 | √3/2 | √3/2 | √3/2 | √3/2 | √3/2 | √3/2 |
| CAPTEUR | 25 |  |  |  |  |  |  |  |
| X | 0 |  |  |  |  |  |  |  |
| Y | 0 |  |  |  |  |  |  |  |
| Z | 1 |  |  |  |  |  |  |  |

**Figure S3: Section of the dome with the magnetic sensors and the detection of the sensors corresponding vectors.** A. Dome, magnetic sensors, vectors and central nucleus with its magnet. B. grouped vectors at a point for calculations. C. Table of Cartesian coordinates of each magnetic sensor.


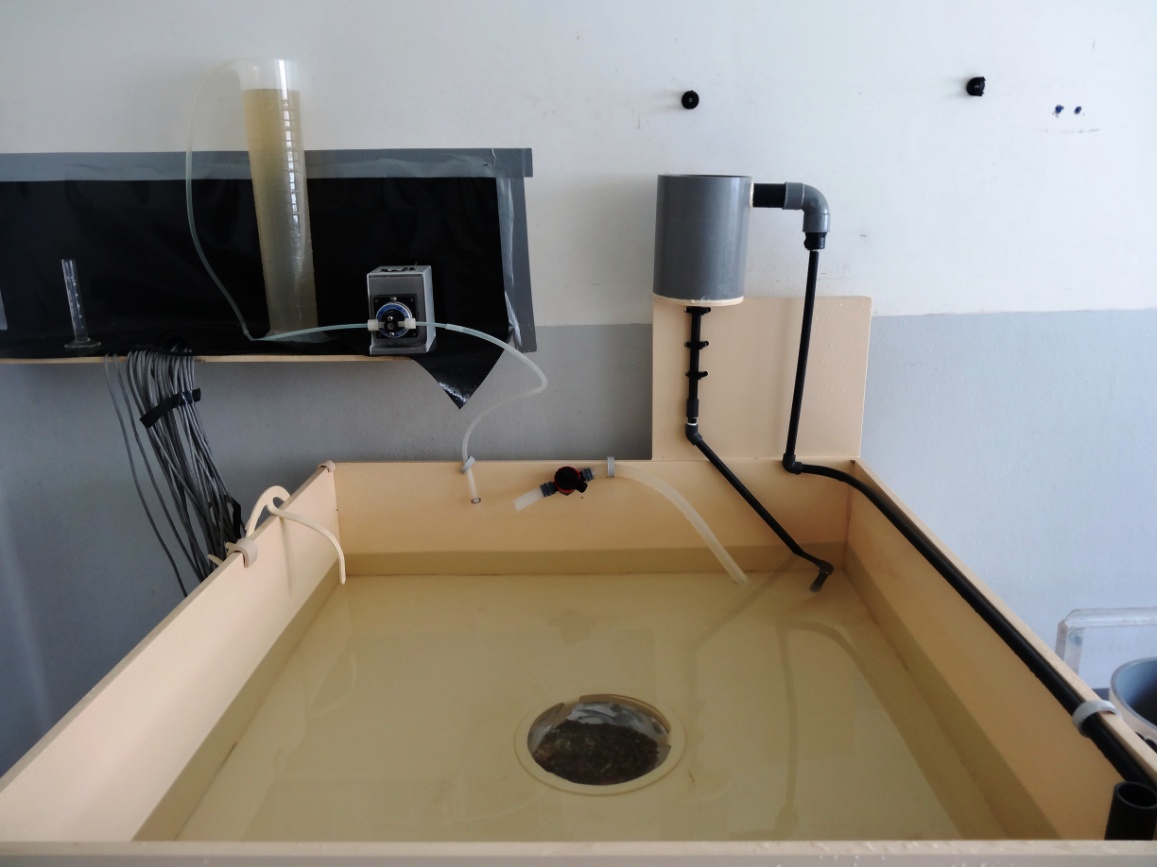


**5**

**A**

**B**

**4**

**3**

**2**

**1**


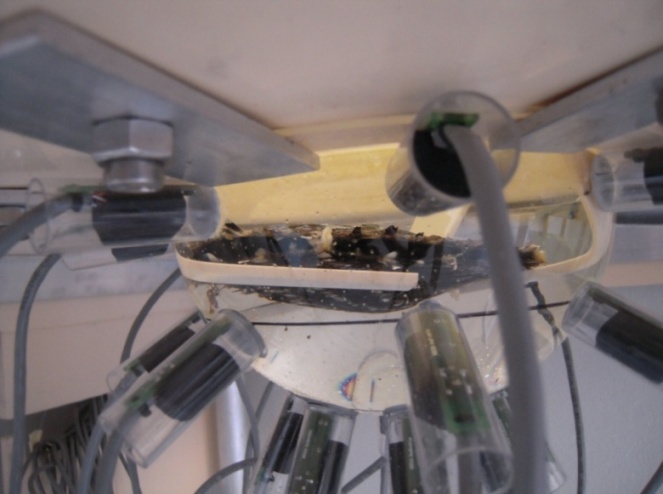


**Figure S4: Illustration of the data acquisition system. A.** Overview of the whole system. 1- Main rearing tank. 2- Dome inside the oyster. 3- Sensors connecting to the computer. 4- Pump for microalgae. 5- Microalgae. **B.** Focus on the dome with the oyster under the main rearing tank.


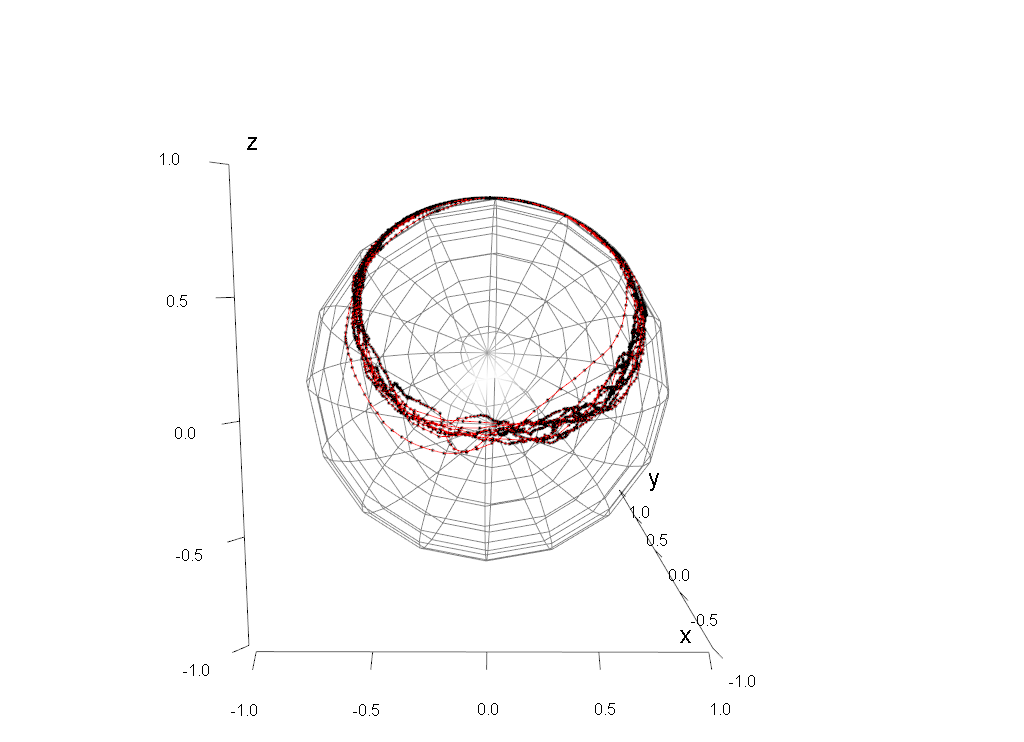


**A B**


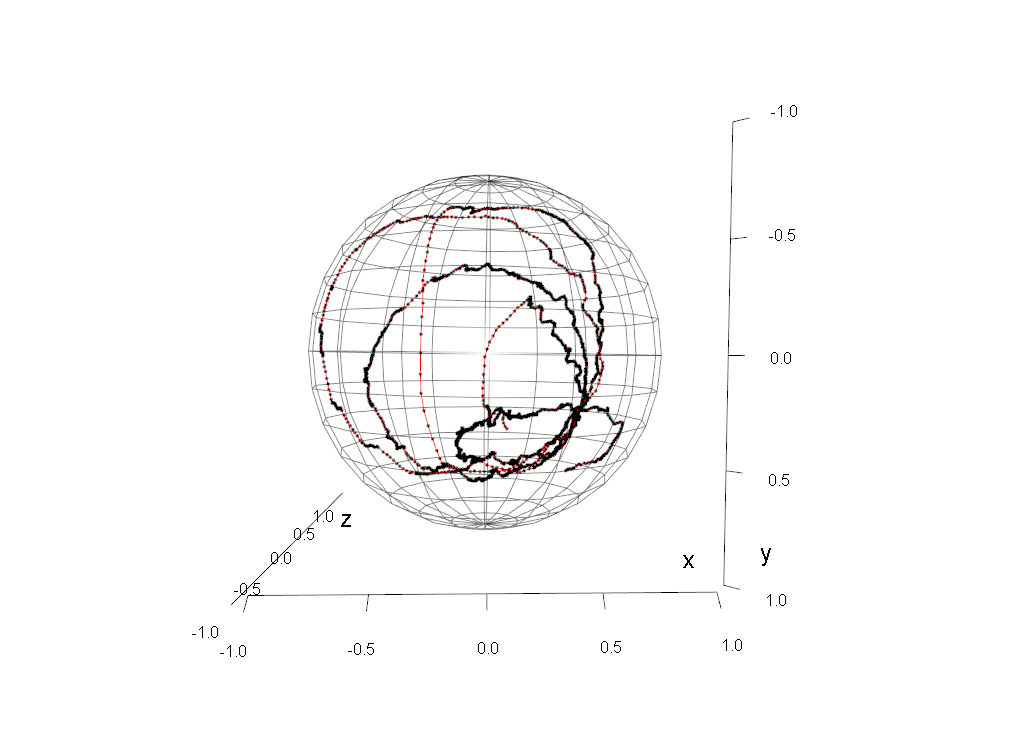


**Figure S5: Contrasted movements of nucleus from two different grafted oysters on a same recording duration (2500 minutes).** (A) Oyster 3. (B) Oyster 4. Points representing measure of magnet position each minute are linked to red lines to show evolution with time.

**
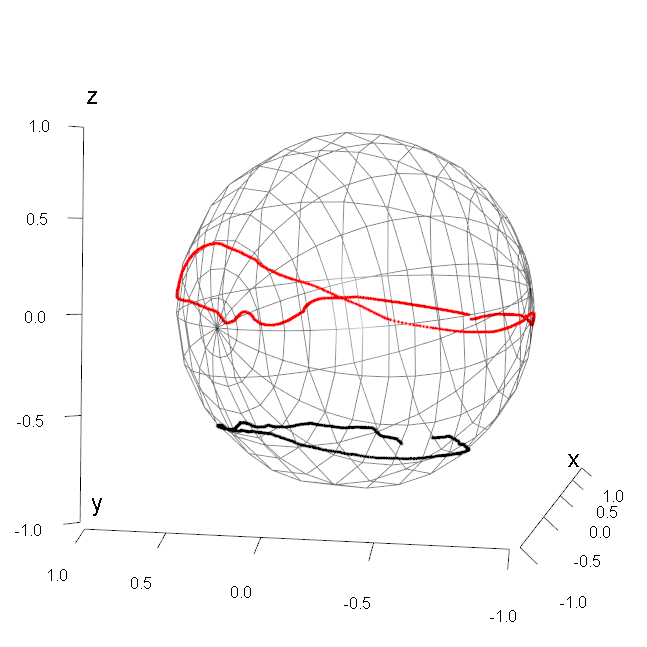
A**  **B**


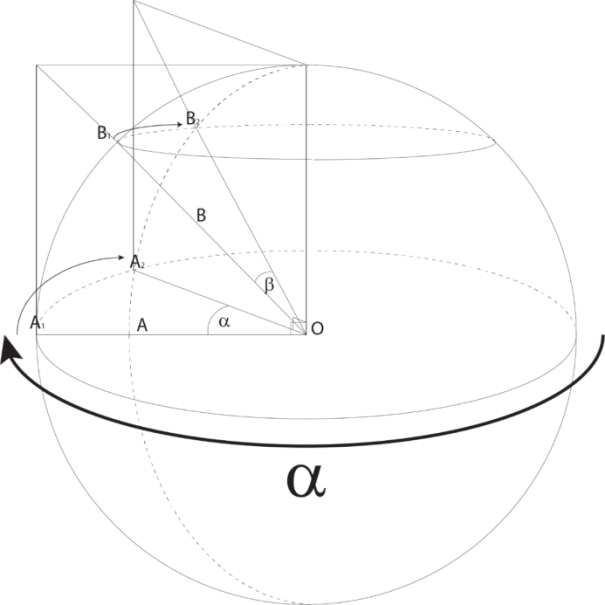


**Figure S6: A) Schema representing different angles of rotation according to angles of the magnet and B) representation of nucleus movement during 931 minutes before (in black) and after (in red) projections, for calculations.** During records, the magnet can be more or less close to the sphere pole of rotation. According to the magnet position, a same rotation, i.e. around a vertical axis (A1 to A2 or B1 to B2), can produce different results without projection, a magnet on the sphere equator giving a measured rotation angle (α) superior to that (β) of a magnet closer to the sphere pole.

**A**


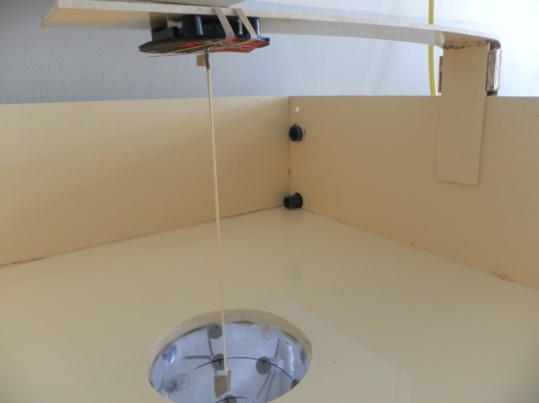


**
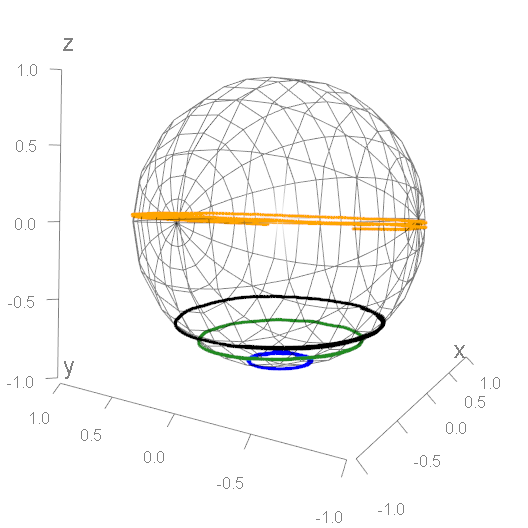

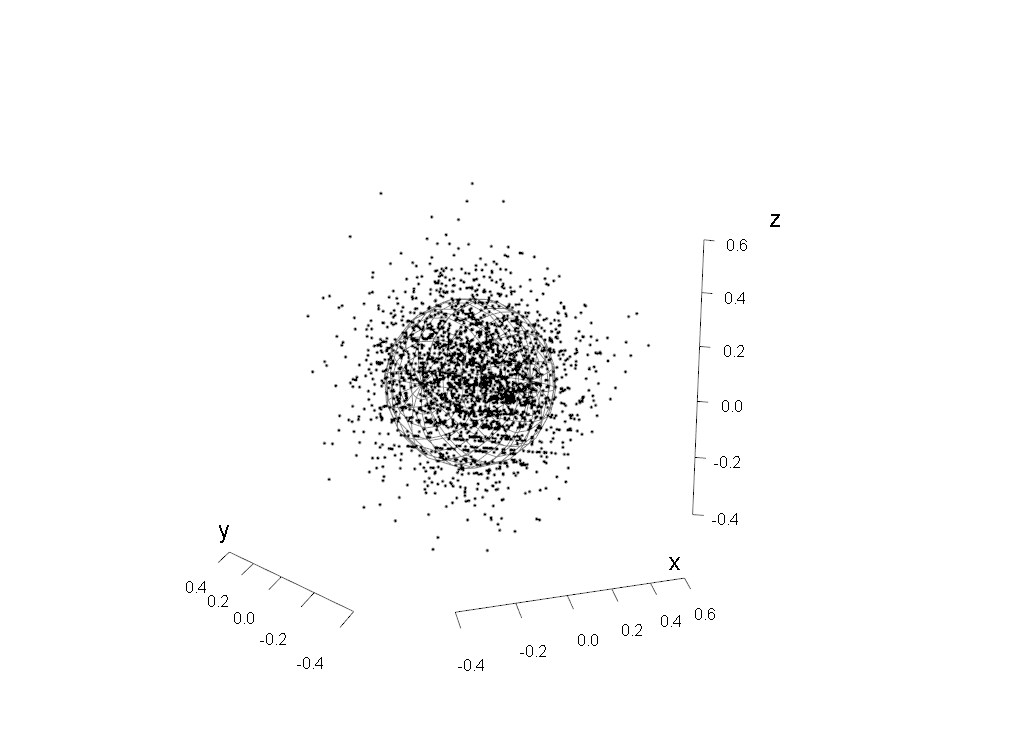
BC**

**Figure S7: Calibration of the « magnetometer » measurement system. (a)** A clock mechanism was used to get a constant rotation with a known speed. The clock system was put up to the monitoring dome with a stick glued to the minute hand mechanism. The magnet is glued on the other end of the stick and positioned in the monitoring area of the dome. The magnet was placed from 0° to 90° (perpendicular) to the stick axis with the following angle in between. **(b)** Three dimensional representations of positions for rotating magnets fixed to the stick placed at 90° (orange), 90° (slanted magnet, green), 60° (black) and 30° (blue)to the horizontal after filtering and **(c)** for motionless magnet before filtering.
